# Supplementary material for: Spatiotemporal variations in retrovirus-host interactions among Darwin’s finches
Source: Nat Commun. 2022 Oct 13;13:6033. doi: 10.1038/s41467-022-33723-w (PMC9562234; doi:10.1038/s41467-022-33723-w)
Supplement: Supplementary file 1 — Supplementary Information [file 41467_2022_33723_MOESM1_ESM.pdf]

Supplementary information for

Spatiotemporal variations in retrovirus-host interactions among Darwin's finches

Jason Hill[1#*], Mette Lillie[1#*], Mats E Pettersson[1], Carl-Johan Rubin[1,2], B Rosemary Grant[3], Peter R Grant[3], Leif Andersson[1,4,5] and Patric Jern[1*]

[1]*Science for Life Laboratory, Department of Medical Biochemistry and Microbiology, Uppsala University, SE-751 23, Uppsala, Sweden*

[2]*Institute of Marine Research, P.O. Box 1870, Nordnes, NO-5817, Bergen, Norway*

[3]*Department of Ecology & Evolutionary Biology, Princeton University, Princeton, NJ 08544, USA*

[4]*Department of Animal Breeding and Genetics, Swedish University of Agricultural Sciences, SE-750 07, Uppsala, Sweden*

[5]*Department of Veterinary Integrative Biosciences, Texas A&M University, College Station, TX 77843, USA*

[#]Equal contributions

*Correspondence:

Jason Hill	Jason.Hill@imbim.uu.se
Mette Lillie	Mette.Lillie@imbim.uu.se
Patric Jern	Patric.Jern@imbim.uu.se

This PDF file includes:

Supplementary Fig. 1 – 6
Supplementary Table 1 – 2

Other supplementary materials for this manuscript include the following:

Supplementary Data 1 – 2

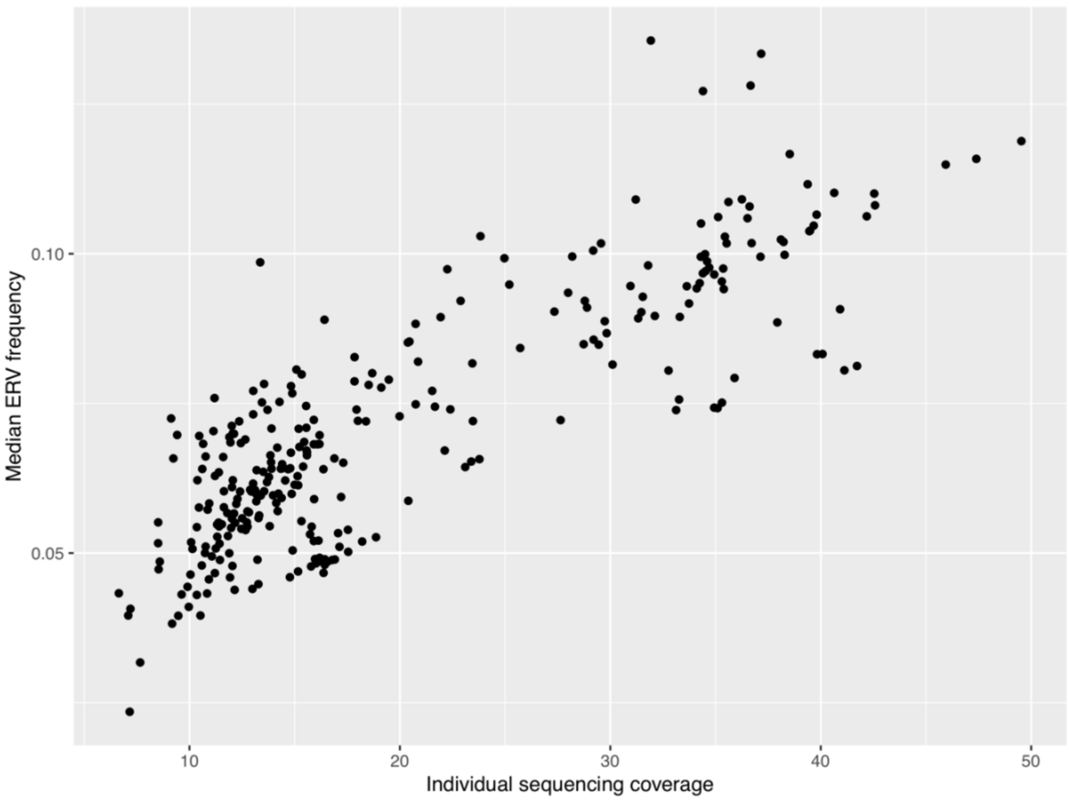

Supplementary Fig. 1 | Relationship between ERV detection by RetroSeq and mapped coverage. Plot of mean whole-genome sequencing mapped coverage (x) and the number of detected RetroSeq ERV loci called per individual.

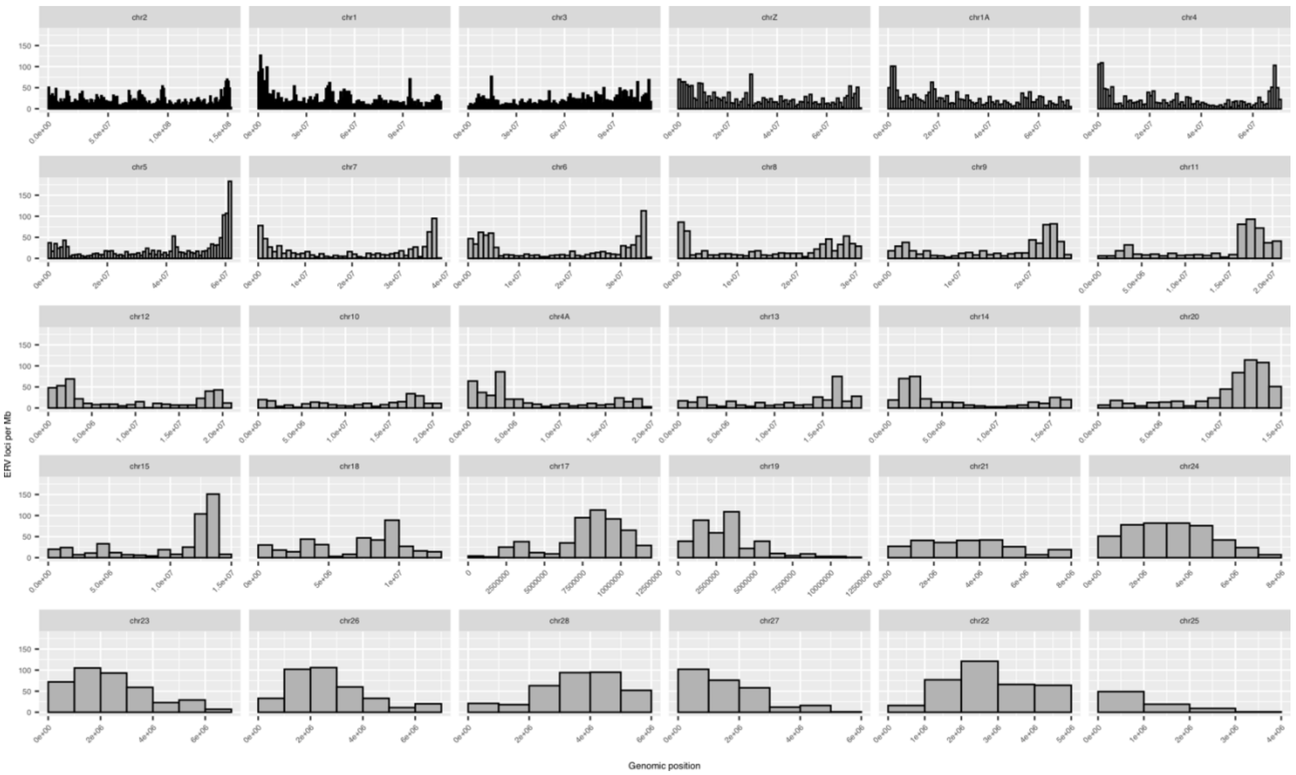

Supplementary Fig. 2 | ERV genome density by chromosomes. Non-uniform overall ERV distribution density histograms showing differences between and along chromosomes.

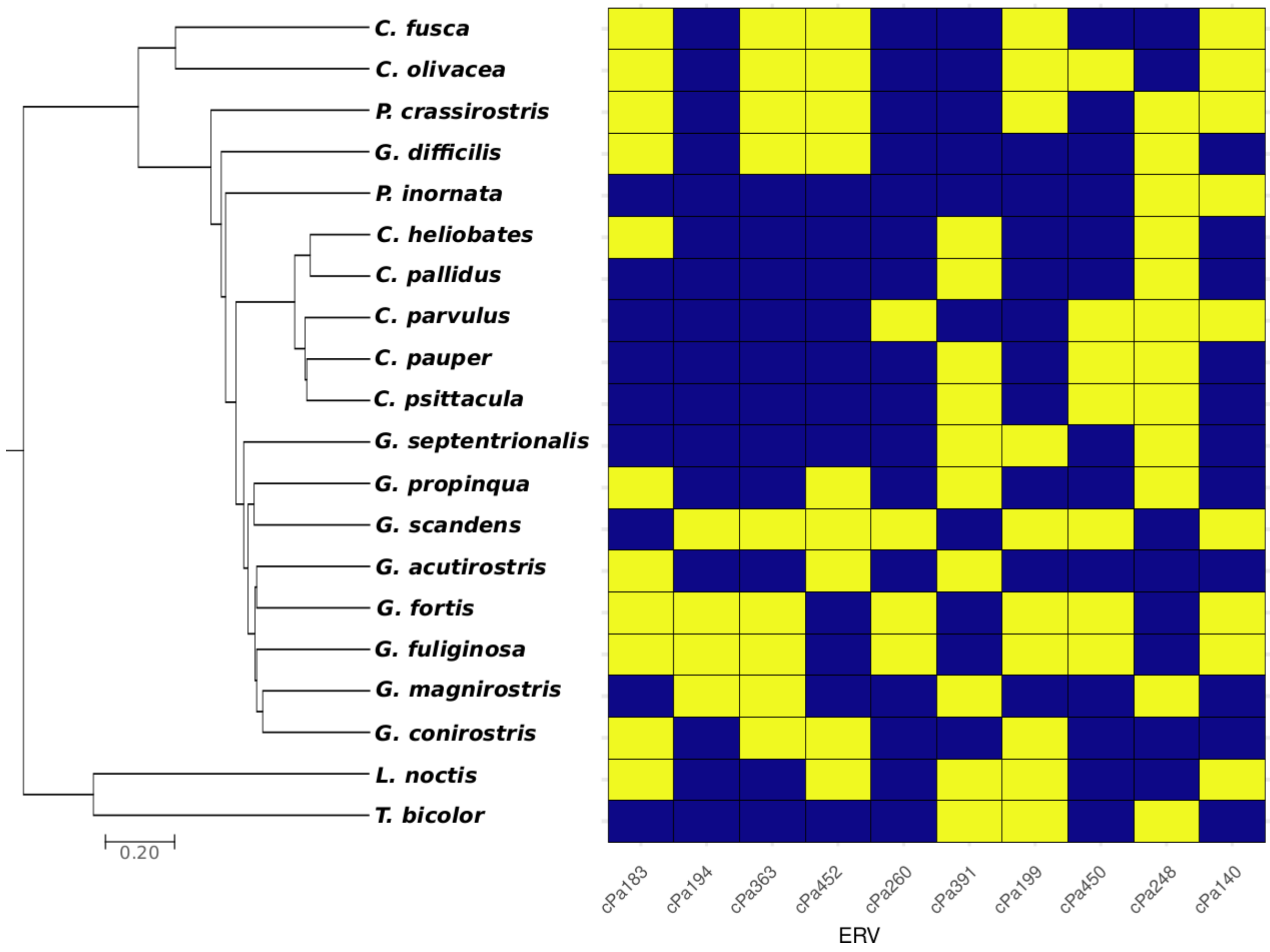

Supplementary Fig. 3 | ERV enrichment in host phylogenetic context. The enrichment of each of the top 10 most abundant ERVs is shown for the investigated non-hybrid species of Darwin's finch. An MIR value > 1 (yellow cell) indicates that an ERV is more abundant within a given species than the median of abundance of across all species. An MIR value < 1 (blue cell) indicates that a given species is deficient in a particular ERV. In general, the distribution of enrichment in the top 10 most abundant ERVs does not follow an obvious pattern, and the same holds true when all ERVs are considered, indicating that no single species or clade of Darwin's finches are relatively enriched for closely related ERVs.

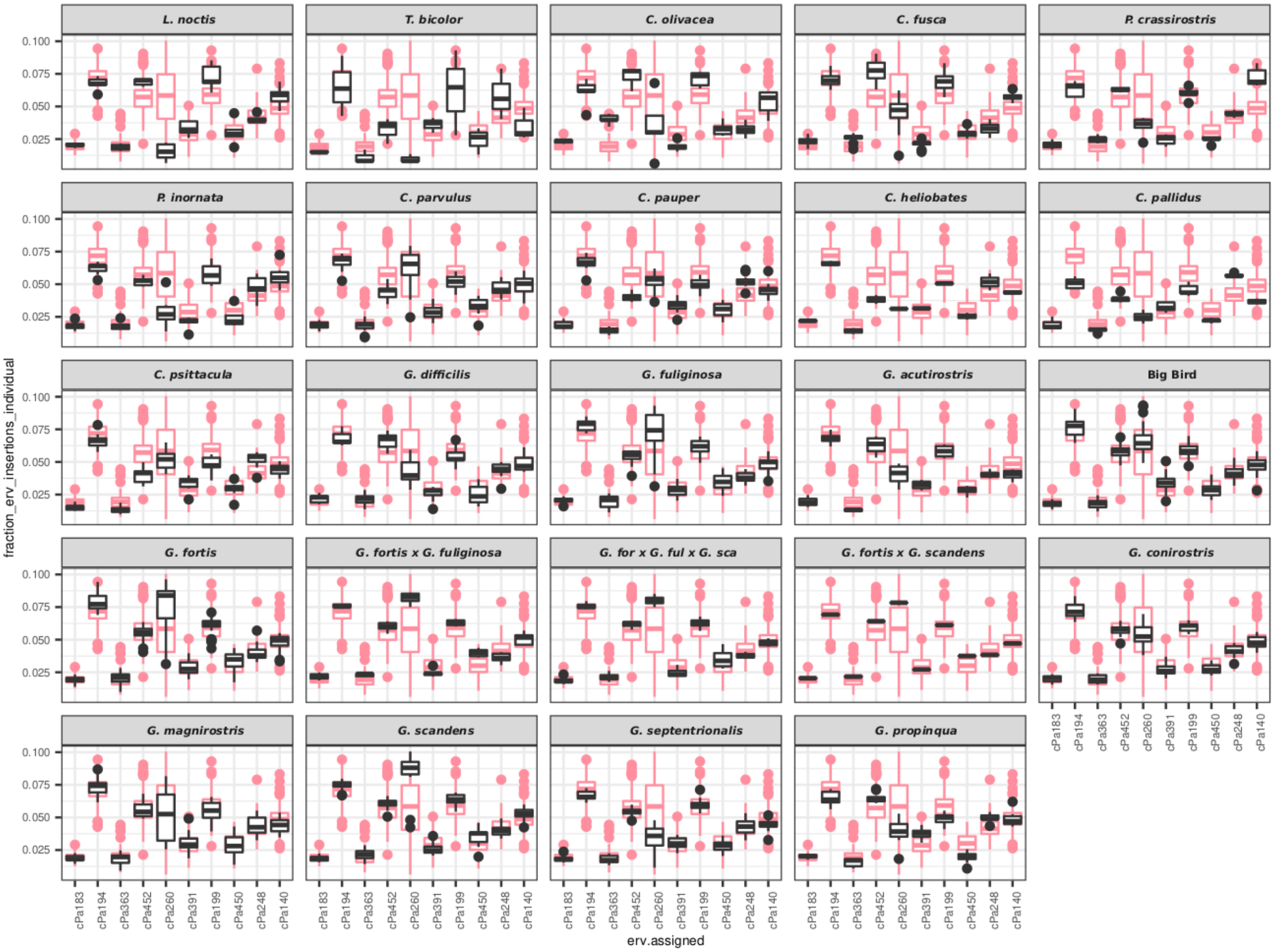

Supplementary Fig. 4 | Relative abundance of common ERVs. The 10 most frequent ERVs showed significant variation in abundance both within and between species. The relative fraction of insertions of an ERV within an individual was plotted as a data point in the box plots. Red data points are for all finch samples, and black is the subset corresponding to only the species in the labeled window. The boxplot bottom and top hinges represent 25% and 75% confidence respectively, and whiskers indicate the 95% confidence interval. Variation in relative ERV abundance among all samples (e.g. cPa260), could be attributed to either within species variation (e.g. *G. fortis* and *G. magnirostris*), between species variation (e.g. *L. noctis* and *G. scandens*), or both.

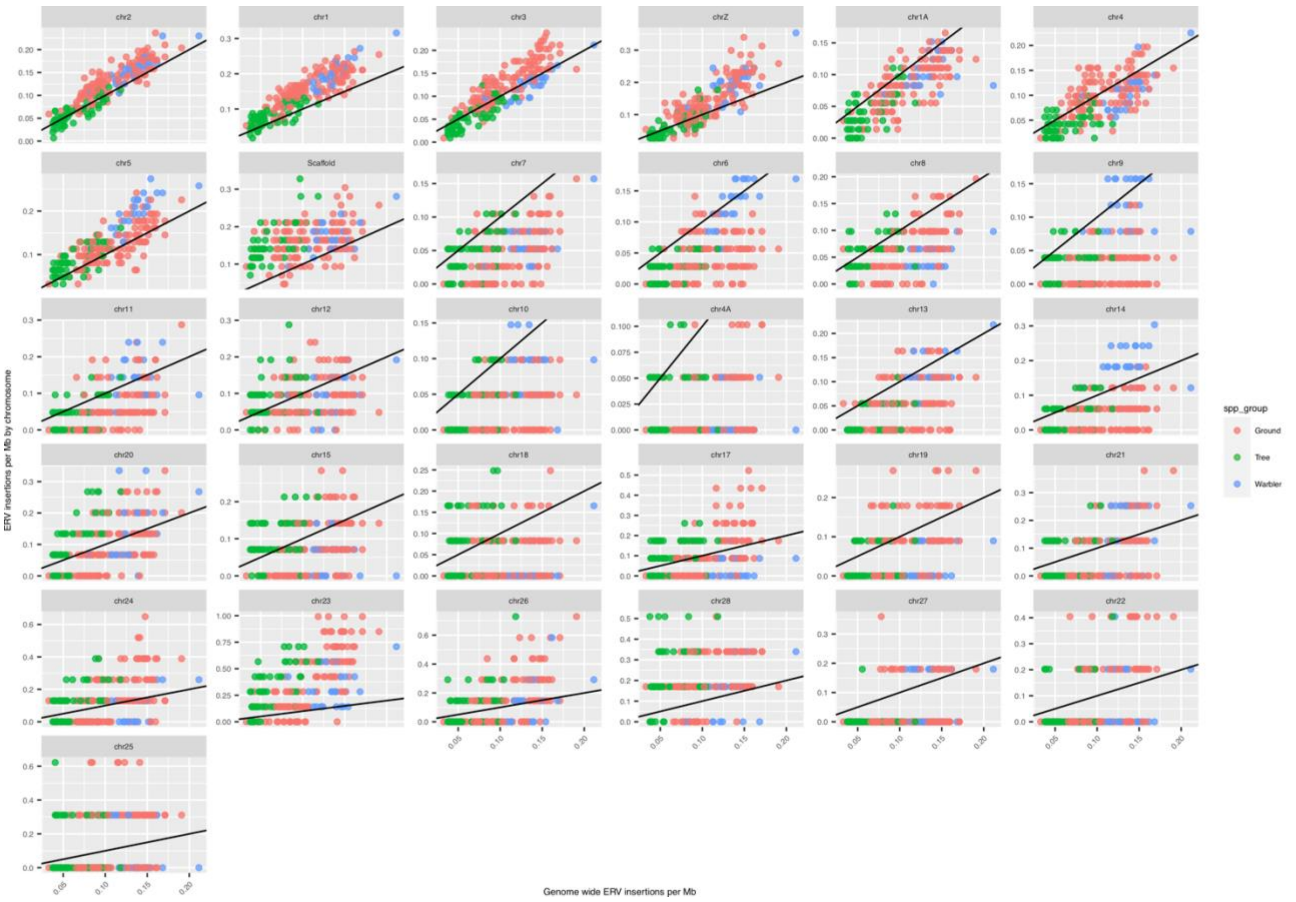

Supplementary Fig. 5 | Density of cPa452 by chromosome for warbler-, tree- and ground finches. Density on each chromosome of *Beta-like 1* ERV cPa452 compared to overall density for each individual sample of ground finch (red color), tree finch (green color), and warbler finch (blue color). Points above the line indicate that cPa452 occurs with a higher density on the chromosome indicated than in the overall genome, where density is measured in ERVs per Mb.

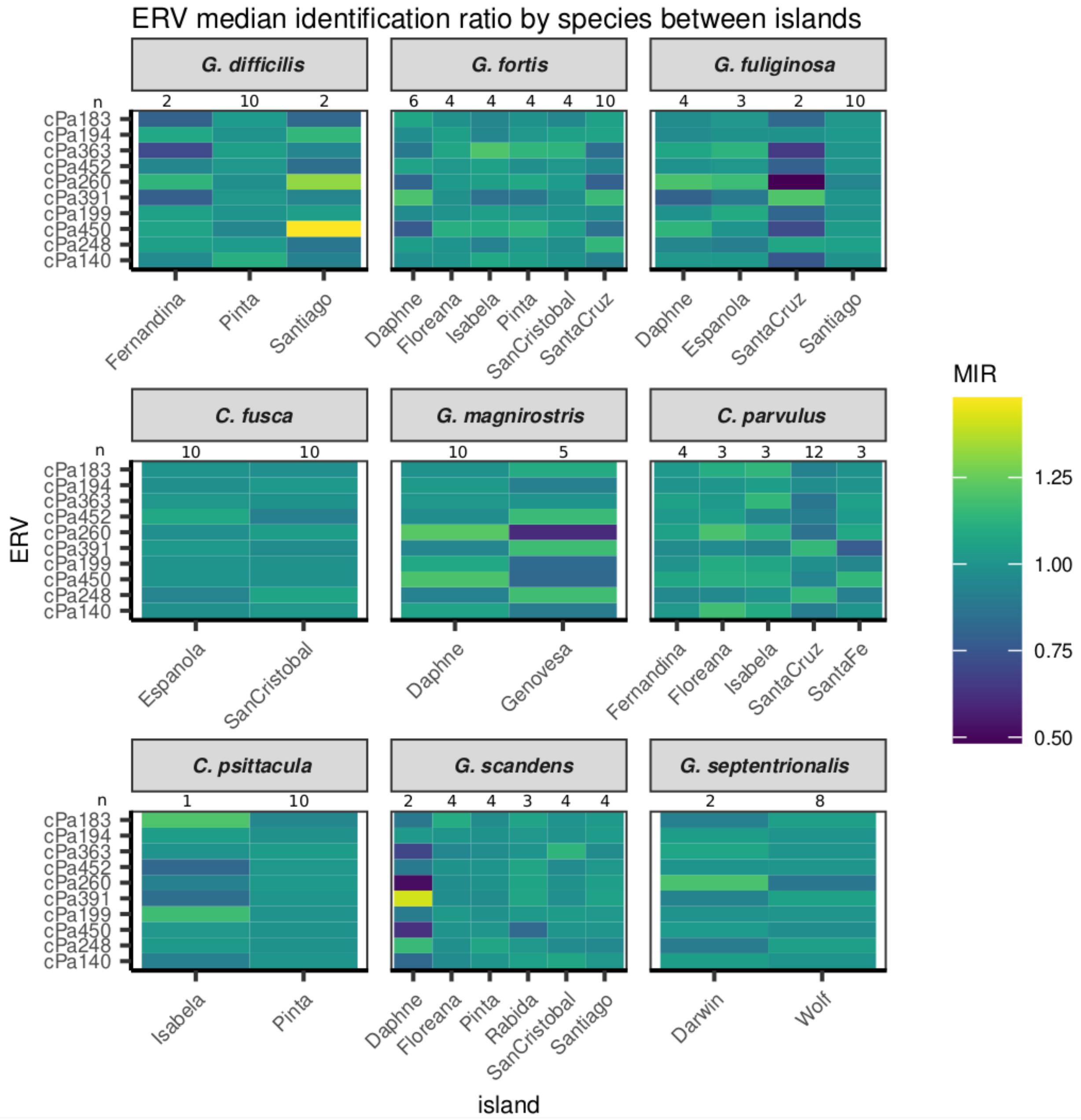

Supplementary Fig. 6 | Contrasting ERV landscapes across finches and islands. Relative abundance of the 10 most frequent ERVs showed variation between island populations of the same species. Modified MIR normalized ERV abundance values both vertically across ERVs and horizontally across islands. A contrasting color between island populations for a given ERV indicates a difference in relative abundance between populations, either higher (lighter color), or lower (darker color). Variation in MIR increased with small sample size (e.g. *G. scandens* from Daphne island), however some contrasts are more likely to represent large actual differences in ERV abundance between island populations (e.g. cPa260 in *G. magnirostris*).

Supplementary Table 1 | Nomenclature conversion.
Nomenclature conversion for phylogeny taxa and locus specific identifiers where "X" is a place holder for locus number 1–n.

clade	fasta/phylogeny	dataframe
Beta-like1	cPa219	ERV-AB1.cPa219.X-CamPar
Beta-like1	cPa258	ERV-AB1.cPa258.X-CamPar
Beta-like1	cPa190	ERV-AB1.cPa190.X-CamPar
Beta-like1	cPa220	ERV-AB1.cPa220.X-CamPar
Beta-like1	cPa104	ERV-AB1.cPa104.X-CamPar
Beta-like1	cPa18	ERV-AB1.cPa18.X-CamPar
Beta-like1	cPa541	ERV-AB1.cPa541.X-CamPar
Beta-like1	cPa4	ERV-AB1.cPa4.X-CamPar
Beta-like1	cPa14	ERV-AB1.cPa14.X-CamPar
Beta-like1	cPa95	ERV-AB1.cPa95.X-CamPar
Beta-like1	cPa247	ERV-AB1.cPa247.X-CamPar
Beta-like1	cPa545	ERV-AB1.cPa545.X-CamPar
Beta-like1	cPa529	ERV-AB1.cPa529.X-CamPar
Beta-like1	cPa479	ERV-AB1.cPa479.X-CamPar
Beta-like1	cPa478	ERV-AB1.cPa478.X-CamPar
Beta-like1	cPa255	ERV-AB1.cPa255.X-CamPar
Beta-like1	cPa131	ERV-AB1.cPa131.X-CamPar
Beta-like1	cPa216	ERV-AB1.cPa216.X-CamPar
Beta-like1	cPa150	ERV-AB1.cPa150.X-CamPar
Beta-like1	cPa302	ERV-AB1.cPa302.X-CamPar
Beta-like1	cPa246	ERV-AB1.cPa246.X-CamPar
Beta-like1	cPa526	ERV-AB1.cPa526.X-CamPar
Beta-like1	cPa463	ERV-AB1.cPa463.X-CamPar
Beta-like1	cPa563	ERV-AB1.cPa563.X-CamPar
Beta-like1	cPa549	ERV-AB1.cPa549.X-CamPar
Beta-like1	cPa523	ERV-AB1.cPa523.X-CamPar
Beta-like1	cPa387	ERV-AB1.cPa387.X-CamPar
Beta-like1	cPa527	ERV-AB1.cPa527.X-CamPar
Beta-like1	cPa395	ERV-AB1.cPa395.X-CamPar
Beta-like1	cPa60	ERV-AB1.cPa60.X-CamPar
Beta-like1	cPa285	ERV-AB1.cPa285.X-CamPar
Beta-like1	cPa546	ERV-AB1.cPa546.X-CamPar
Beta-like1	cPa257	ERV-AB1.cPa257.X-CamPar
Beta-like1	cPa130	ERV-AB1.cPa130.X-CamPar
Beta-like1	cPa472	ERV-AB1.cPa472.X-CamPar
Beta-like1	cPa575	ERV-AB1.cPa575.X-CamPar
Beta-like1	cPa572	ERV-AB1.cPa572.X-CamPar
Beta-like1	cPa518	ERV-AB1.cPa518.X-CamPar
Beta-like1	cPa138	ERV-AB1.cPa138.X-CamPar
Beta-like1	cPa510	ERV-AB1.cPa510.X-CamPar
Beta-like1	cPa200	ERV-AB1.cPa200.X-CamPar
Beta-like1	cPa544	ERV-AB1.cPa544.X-CamPar
Beta-like1	cPa542	ERV-AB1.cPa542.X-CamPar
Beta-like1	cPa177	ERV-AB1.cPa177.X-CamPar
Beta-like1	cPa515	ERV-AB1.cPa515.X-CamPar
Beta-like1	cPa540	ERV-AB1.cPa540.X-CamPar
Beta-like1	cPa241	ERV-AB1.cPa241.X-CamPar
Beta-like1	cPa127	ERV-AB1.cPa127.X-CamPar
Beta-like1	cPa530	ERV-AB1.cPa530.X-CamPar
Beta-like1	cPa11	ERV-AB1.cPa11.X-CamPar
Beta-like1	cPa290	ERV-AB1.cPa290.X-CamPar
Beta-like1	cPa552	ERV-AB1.cPa552.X-CamPar
Beta-like1	cPa555	ERV-AB1.cPa555.X-CamPar
Beta-like1	cPa543	ERV-AB1.cPa543.X-CamPar
Beta-like1	cPa101	ERV-AB1.cPa101.X-CamPar
Beta-like1	cPa256	ERV-AB1.cPa256.X-CamPar
Beta-like1	cPa142	ERV-AB1.cPa142.X-CamPar
Beta-like1	cPa582	ERV-AB1.cPa582.X-CamPar
Beta-like1	cPa551	ERV-AB1.cPa551.X-CamPar
Beta-like1	cPa51	ERV-AB1.cPa51.X-CamPar
Beta-like1	cPa511	ERV-AB1.cPa511.X-CamPar
Beta-like1	cPa333	ERV-AB1.cPa333.X-CamPar
Beta-like1	cPa50	ERV-AB1.cPa50.X-CamPar
Beta-like1	cPa183	ERV-AB1.cPa183.X-CamPar
Beta-like1	cPa500	ERV-AB1.cPa500.X-CamPar

Beta-like1	cPa34	ERV-AB1.cPa34.X-CamPar
Beta-like1	cPa188	ERV-AB1.cPa188.X-CamPar
Beta-like1	cPa194	ERV-AB1.cPa194.X-CamPar
Beta-like1	cPa74	ERV-AB1.cPa74.X-CamPar
Beta-like1	cPa402	ERV-AB1.cPa402.X-CamPar
Beta-like1	cPa585	ERV-AB1.cPa585.X-CamPar
Beta-like1	cPa400	ERV-AB1.cPa400.X-CamPar
Beta-like1	cPa394	ERV-AB1.cPa394.X-CamPar
Beta-like1	cPa482	ERV-AB1.cPa482.X-CamPar
Beta-like1	cPa173	ERV-AB1.cPa173.X-CamPar
Beta-like1	cPa16	ERV-AB1.cPa16.X-CamPar
Beta-like1	cPa71	ERV-AB1.cPa71.X-CamPar
Beta-like1	cPa176	ERV-AB1.cPa176.X-CamPar
Beta-like1	cPa363	ERV-AB1.cPa363.X-CamPar
Beta-like1	cPa452	ERV-AB1.cPa452.X-CamPar
Beta-like1	cPa446	ERV-AB1.cPa446.X-CamPar
Beta-like1	cPa379	ERV-AB1.cPa379.X-CamPar
Beta-like1	cPa486	ERV-AB1.cPa486.X-CamPar
Beta-like1	cPa381	ERV-AB1.cPa381.X-CamPar
Beta-like1	cPa390	ERV-AB1.cPa390.X-CamPar
Beta-like2	cPa327	ERV-AB2.cPa327.X-CamPar
Beta-like2	cPa424	ERV-AB2.cPa424.X-CamPar
Beta-like2	cPa459	ERV-AB2.cPa459.X-CamPar
Beta-like2	cPa196	ERV-AB2.cPa196.X-CamPar
Beta-like2	cPa308	ERV-AB2.cPa308.X-CamPar
Beta-like2	cPa433	ERV-AB2.cPa433.X-CamPar
Beta-like2	cPa305	ERV-AB2.cPa305.X-CamPar
Beta-like2	cPa260	ERV-AB2.cPa260.X-CamPar
Beta-like2	cPa521	ERV-AB2.cPa521.X-CamPar
Beta-like2	cPa228	ERV-AB2.cPa228.X-CamPar
Beta-like2	cPa502	ERV-AB2.cPa502.X-CamPar
Beta-like2	cPa353	ERV-AB2.cPa353.X-CamPar
Beta-like2	cPa391	ERV-AB2.cPa391.X-CamPar
Beta-like2	cPa426	ERV-AB2.cPa426.X-CamPar
Beta-like2	cPa313	ERV-AB2.cPa313.X-CamPar
Beta-like2	cPa332	ERV-AB2.cPa332.X-CamPar
Beta-like2	cPa157	ERV-AB2.cPa157.X-CamPar
Beta-like2	cPa199	ERV-AB2.cPa199.X-CamPar
Beta-like	HML7_repbase_HERVK11DI_MER11D	ERV-K(HML7).X-CamPar
Beta-like	HML8_repbase_HERVK11I_MER11A	ERV-K(HML8).X-CamPar
Beta-like	HML2_Pheonix	ERV-Phoenix.X-CamPar
Beta-like	HML2_repbase_HERVK_LTR5	ERV-K(HML2).X-CamPar
Beta-like	HML1a_repbase_HERV-K14CI_LTR14C	ERV-K(HML1a).X-CamPar
Beta-like	HML1b_repbase_HERV-K14I_LTR14A	ERV-K(HML1b).X-CamPar
Beta-like	HML4_repbase_HERVK13I_LTR13	ERV-K(HML4).X-CamPar
Beta-like	HML3_repbase_HERVK9I_MER9	ERV-K(HML3).X-CamPar
Beta-like	HML5_repbase_HERVK22I_LTR22	ERV-K(HML5).X-CamPar
Beta-like	HML6_repbase_HERVK3I_LTR3	ERV-K(HML6).X-CamPar
Beta	ENTV_NC004994	ERV-ENTV.X-CamPar
Beta	JSRV_DQ838494	ERV-JSRV.X-CamPar
Beta	MPMVs_NC001550	ERV-MPMV.X-CamPar
Beta	SMRV_NC001514	ERV-SMRV.X-CamPar
Beta	MMTV_NC001503	ERV-MMTV.X-CamPar
Beta	PyERV_AF500296	ERV-PyERV.X-CamPar
Beta-like	cPa492	ERV-AB.cPa492.X-CamPar
Alpha	ALVA_M37980	ERV-ALVA.X-CamPar
Alpha	ALV_NC001408	ERV-ALV.X-CamPar
Alpha	ALVE_FJ793550	ERV-ALVE.X-CamPar
Alpha	ALVJ_Z46390	ERV-ALVJ.X-CamPar
Alpha-like	cPa224	ERV-AB.cPa224.X-CamPar
Alpha-like	cPa360	ERV-AB.cPa360.X-CamPar
Alpha-like	cPa163	ERV-AB.cPa163.X-CamPar
Alpha-like	cPa182	ERV-AB.cPa182.X-CamPar
Lenti	SIV_NC001870	ERV-SIV.X-CamPar
Lenti	HIV2_NC001722	ERV-HIV2.X-CamPar
Lenti	HIV1_NC001802	ERV-HIV1.X-CamPar
Lenti	SIV_NC001549	ERV-SIV.X-CamPar
Lenti	RELIK_ERVK	ERV-RELIK.X-CamPar
Lenti	EIAV_NC001450	ERV-EIAV.X-CamPar
Lenti	FIV_NC001482	ERV-FIV.X-CamPar
Lenti	VISNA_S55323	ERV-VISNA.X-CamPar
Lenti	CAEV_NC001463	ERV-CAEV.X-CamPar
Lenti	BIV_NC001413	ERV-BIV.X-CamPar

Delta	HTLV1_NC001436	ERV-HTLV1.X-CamPar
Delta	STLV1_NC000858	ERV-STLV1.X-CamPar
Delta	STLV2_NC001815	ERV-STLV2.X-CamPar
Delta	HTLV2_M10060	ERV-HTLV2.X-CamPar
Delta	BLV_NC001414	ERV-BLV.X-CamPar
Gamma	PERVA_AY099323	ERV-PERVA.X-CamPar
Gamma	PERVB_AY099324	ERV-PERVB.X-CamPar
Gamma	PERVC_HM159246	ERV-PERVC.X-CamPar
Gamma	PERV_AJ293656	ERV-PERV.X-CamPar
Gamma	KWERV_GQ222416	ERV-KWERV.X-CamPar
Gamma	MDEV_AF053745	ERV-MDEV.X-CamPar
Gamma	MSEV_CT990572	ERV-MSEV.X-CamPar
Gamma	KoRV_AF151794	ERV-KoRV.X-CamPar
Gamma	GaLV_M26927	ERV-GaLV.X-CamPar
Gamma	MoMLV_J02255	ERV-MoMLV.X-CamPar
Gamma	FrMuLV_NC001362	ERV-FrMuLV.X-CamPar
Gamma	MLV_Mpmv5	ERV-Mpmv.5.X-CamPar
Gamma	MLV_Pmv4	ERV-Pmv.4.X-CamPar
Gamma	MLV_Xmv43	ERV-Xmv.43.X-CamPar
Gamma	FeLV_NC001940	ERV-FeLV.X-CamPar
Gamma	BaEV_D10032	ERV-BaEV.X-CamPar
Gamma	PtERV1_AC142352	ERV-PtERV1.X-CamPar
Gamma	rRVRV_JQ303225	ERV-rRVRV.X-CamPar
Gamma	REV_NC006934	ERV-REV.X-CamPar
Gamma-like	S71_repbase_HERVS71_LTR6A	ERV-S71.X-CamPar
Gamma-like	HERV-E_repbase_HERVE_LTR2	ERV-HERVE.X-CamPar
Gamma-like	PERVE_NC003059	ERV-PERVE.X-CamPar
Gamma-like	HERV-R_repbase_HERV3_LTR4	ERV-HERVR.X-CamPar
Gamma-like	HERV-XA34_repbase_HERVFH21I_LTR21A	ERV-HERVXA34.X-CamPar
Gamma-like	HERVFc2_repbase	ERV-HERVFc2.X-CamPar
Gamma-like	HERVHcons	ERV-GE.cPaHcons.X-CamPar
Gamma-like	HERV-Fb_repbase_HERVH48I_MER48	ERV-HERVFb.X-CamPar
Gamma-like	HERV-F_repbase_HERVFH19I_LTR19A	ERV-HERVF.X-CamPar
Gamma-like	HERV30_repbase_HERV30I_LTR30	ERV-HERV30.X-CamPar
Gamma-like	HERV-W_repbase_HERV17_LTR17	ERV-HERVW.X-CamPar
Gamma-like	MER52A_repbase_MER52AI_MER52A	ERV-MER52A.X-CamPar
Gamma-like	PRIMA41_repbase_PRIMA41_MER41A	ERV-PRIMA41.X-CamPar
Gamma-like	cPa358	ERV-GE.cPa358.X-CamPar
Gamma-like	cPa251	ERV-GE.cPa251.X-CamPar
Gamma-like	cPa113	ERV-GE.cPa113.X-CamPar
Gamma-like	cPa447	ERV-GE.cPa447.X-CamPar
Gamma-like	cPa227	ERV-GE.cPa227.X-CamPar
Gamma-like	cPa357	ERV-GE.cPa357.X-CamPar
Gamma-like	cPa273	ERV-GE.cPa273.X-CamPar
Gamma-like	cPa583	ERV-GE.cPa583.X-CamPar
Gamma-like	cPa168	ERV-GE.cPa168.X-CamPar
Gamma-like	cPa450	ERV-GE.cPa450.X-CamPar
Gamma-like	cPa475	ERV-GE.cPa475.X-CamPar
Gamma-like	cPa469	ERV-GE.cPa469.X-CamPar
Gamma-like	cPa62	ERV-GE.cPa62.X-CamPar
Gamma-like	cPa201	ERV-GE.cPa201.X-CamPar
Gamma-like	cPa505	ERV-GE.cPa505.X-CamPar
Gamma-like	cPa152	ERV-GE.cPa152.X-CamPar
Gamma-like	cPa109	ERV-GE.cPa109.X-CamPar
Gamma-like	cPa205	ERV-GE.cPa205.X-CamPar
Gamma-like	cPa61	ERV-GE.cPa61.X-CamPar
Gamma-like	HERV-I_repbase_HERVI_LTR10B	ERV-HERVI.X-CamPar
Gamma-like	HERV-ADP_repbase_HERVP71A_I_LTR71A	ERV-HERVADP.X-CamPar
Epsilon	WDSV_NC001867	ERV-WDSV.X-CamPar
Epsilon	Xen1_AJ506107	ERV-Xen1.X-CamPar
SnRV-like	cPa443	ERV-S.cPa443.X-CamPar
SnRV-like	cPa264	ERV-S.cPa264.X-CamPar
SnRV-like	cPa15	ERV-S.cPa15.X-CamPar
SnRV-like	cPa248	ERV-S.cPa248.X-CamPar
SnRV-like	cPa140	ERV-S.cPa140.X-CamPar
SnRV-like	SnRV_NC001724	ERV-SnRV.X-CamPar
Spuma	EqFV_NC002201	ERV-EqFV.X-CamPar
Spuma	BFV_NC001831	ERV-BFV.X-CamPar
Spuma	FeFV_NC001871	ERV-FeFV.X-CamPar
Spuma	HFV_NC001736	ERV-HFV.X-CamPar
Spuma	SFV_U04327	ERV-SFV.X-CamPar
Spuma	MSFV_NC010819	ERV-MSFV.X-CamPar
Gypsy	Cer1_U15406	Gypsy-Cer1.X-CamPar

Supplementary Table 2 | ERV locus density by chromosome.

chromosome	number of loci	chromosome size	ERV loci per MB
chr2	3149	152240728	20,68
chr1	2732	113967701	23,97
chr3	2417	113435954	21,31
chrZ	1888	73580794	25,66
chr1A	1708	72438334	23,58
chr4	1567	71061686	22,05
chr5	1389	62010874	22,40
chr7	699	38189422	18,30
chr6	751	35412503	21,21
chr8	644	30584378	21,06
chr9	560	25423666	22,03
chr11	487	20882383	23,32
chr12	417	20836500	20,01
chr10	249	20345023	12,24
chr4A	404	19694112	20,51
chr13	314	18340482	17,12
chr14	327	16472583	19,85
chr20	531	14936690	35,55
chr15	439	14104672	31,12
chr18	383	12083600	31,70
chr17	519	11493945	45,15
chr19	388	11160396	34,77
chr21	239	7910879	30,21
chr24	442	7709400	57,33
chr23	388	7058734	54,97
chr26	365	6857747	53,22
chr28	343	5889000	58,24
chr27	265	5560827	47,65
chr22	344	4946000	69,55
chr25	78	3215879	24,25
