# Supplementary material for: Spatiotemporal variations in retrovirus-host interactions among Darwin’s finches
Source: Nat Commun. 2022 Oct 13;13:6033. doi: 10.1038/s41467-022-33723-w (PMC9562234; doi:10.1038/s41467-022-33723-w)
Supplement: Supplementary file 3 — Description of Additional Supplementary Files [file 41467_2022_33723_MOESM3_ESM.pdf]

Description of Additional Supplementary Files

File Name: Supplementary Data 1
Description: ERV FASTA sequences.

File Name: Supplementary Data 2
Description: ERV nexus tree file.
