# Supplementary material for: Spatiotemporal variations in retrovirus-host interactions among Darwin’s finches
Source: Nat Commun. 2022 Oct 13;13:6033. doi: 10.1038/s41467-022-33723-w (PMC9562234; doi:10.1038/s41467-022-33723-w)
Supplement: Supplementary file 6 — Reporting Summary [file 41467_2022_33723_MOESM6_ESM.pdf]

nature portfolio

Corresponding author(s): Jason Hill
Mette Lille
Patric Jern

Last updated by author(s): Sep 29, 2022

Reporting Summary

Nature Portfolio wishes to improve the reproducibility of the work that we publish. This form provides structure for consistency and transparency in reporting. For further information on Nature Portfolio policies, see our Editorial Policies and the Editorial Policy Checklist.

Statistics

For all statistical analyses, confirm that the following items are present in the figure legend, table legend, main text, or Methods section.

n/a	Confirmed	
☐	☒	The exact sample size (*n*) for each experimental group/condition, given as a discrete number and unit of measurement
☒	☐	A statement on whether measurements were taken from distinct samples or whether the same sample was measured repeatedly
☐	☒	The statistical test(s) used AND whether they are one- or two-sided<br>*Only common tests should be described solely by name; describe more complex techniques in the Methods section.*
☒	☐	A description of all covariates tested
☐	☒	A description of any assumptions or corrections, such as tests of normality and adjustment for multiple comparisons
☐	☒	A full description of the statistical parameters including central tendency (e.g. means) or other basic estimates (e.g. regression coefficient) AND variation (e.g. standard deviation) or associated estimates of uncertainty (e.g. confidence intervals)
☐	☒	For null hypothesis testing, the test statistic (e.g. *F*, *t*, *r*) with confidence intervals, effect sizes, degrees of freedom and *P* value noted<br>*Give P values as exact values whenever suitable.*
☒	☐	For Bayesian analysis, information on the choice of priors and Markov chain Monte Carlo settings
☒	☐	For hierarchical and complex designs, identification of the appropriate level for tests and full reporting of outcomes
☒	☐	Estimates of effect sizes (e.g. Cohen's *d*, Pearson's *r*), indicating how they were calculated

Our web collection on statistics for biologists contains articles on many of the points above.

Software and code

Policy information about availability of computer code

Data collection	No software was used in data analysis
Data analysis	ERV detection: BWA-MEM v0.7.17-intel-2018b, SAMtools v1.14, Picard v1.92, RetroTector v1.0 , FastTree2 v2.1.7, R v3.6.3, GenomicRanges v1.38, CNVnator v0.3.3, DELLY v0.7.7, BLAT v36, BCFtools v1.14, intansv v1.34.0, VariantAnnotation v1.40.0, RetroSeq https://github.com/tk2/RetroSeq (Accessed Feb. 18, 2020)<br><br>ERV segregation analysis: R v4.1.1, Tidyverse v1.3.1, data.table v 1.14.2, ape v 5.2<br><br>Custom R scripts for analysis: https://github.com/PatricJernLab/Darwins_finches_ERV_diversity<br>RetroTector can be found at https://github.com/PatricJernLab/

For manuscripts utilizing custom algorithms or software that are central to the research but not yet described in published literature, software must be made available to editors and reviewers. We strongly encourage code deposition in a community repository (e.g. GitHub). See the Nature Portfolio guidelines for submitting code & software for further information.

Data

Policy information about availability of data

All manuscripts must include a data availability statement. This statement should provide the following information, where applicable:

- Accession codes, unique identifiers, or web links for publicly available datasets
- A description of any restrictions on data availability
- For clinical datasets or third party data, please ensure that the statement adheres to our policy

Data for data analysis: https://github.com/PatricJernLab/Darwins_finches_ERV_diversity
The small tree finch assembly is available at Genbank: GCA_902806625.1
Illumina sequencing data is available from the European Nucleotide Archive under study accession numbers PRJNA743742

Field-specific reporting

Please select the one below that is the best fit for your research. If you are not sure, read the appropriate sections before making your selection.

☐ Life sciences ☐ Behavioural & social sciences ☒ Ecological, evolutionary & environmental sciences

For a reference copy of the document with all sections, see nature.com/documents/nr-reporting-summary-flat.pdf

Ecological, evolutionary & environmental sciences study design

All studies must disclose on these points even when the disclosure is negative.

Study description	Endogenous retrovirus diversity was surveyed within the adaptive radiation of Darwin's finches and two outgroup species. Comparisons were made between species and island populations to estimate ERV allele frequencies.
Research sample	Individual samples of Darwin's finches (n = 285) from Galápagos and Cocos islands, as well as individuals of two species (Loxigilla noctis and Tiaris bicolor) representing outgroups to the ancestor of Darwin's finches sampled from Barbados (n = 8). The samples represent all 18 species of Darwin's finches (n = 226) and four hybrid groups (n = 59). The finch species sampled on Galápagos were further subdivided by populations across 14 of the islands.
Sampling strategy	Sample availability was determined by previously published work by Rosemary Grant and Peter Grant over the course of 40 years. All available samples were used, and normalization procedures were used in analysis to compensate for unequal sample sizes.
Data collection	Blood samples were collected by Peter R. Grant and Rosemary Grant. Short read sequencing libraries were prepared in the laboratory of Leif Andersson. Sequencing data was generated on an Illumina Hiseq2500 by SciLifeLab (Uppsala, Sweden).
Timing and spatial scale	Samples were originally collected in 2013 (All species besides hybrids), 2015 (hybrids), as part of previous studies to investigate beak size morphology, hybridization, and adaptive radiation.
Data exclusions	One locus of 26,963 was removed due to Delly incorrectly assigning an ERV insertion where there was no read support.
Reproducibility	Analysis workflow code is provided with input data for reproduction of results.
Randomization	Samples were allocated into groups based on species and island populations. Randomization was not applied and is not relevant since the purpose of the study was to compare ERV diversity between groups.
Blinding	Blinding was not relevant since there were no subjective observations used as data.

Did the study involve field work? ☐ Yes ☒ No

Reporting for specific materials, systems and methods

We require information from authors about some types of materials, experimental systems and methods used in many studies. Here, indicate whether each material, system or method listed is relevant to your study. If you are not sure if a list item applies to your research, read the appropriate section before selecting a response.

Materials & experimental systems

n/a	Involved in the study
☒	☐ Antibodies
☒	☐ Eukaryotic cell lines
☒	☐ Palaeontology and archaeology
☒	☐ Animals and other organisms
☒	☐ Human research participants
☒	☐ Clinical data
☒	☐ Dual use research of concern

Methods

n/a	Involved in the study
☒	☐ ChIP-seq
☒	☐ Flow cytometry
☒	☐ MRI-based neuroimaging
